# Impaired Recent Verbal Memory in Pornography-Addicted Juvenile Subjects

**DOI:** 10.1155/2019/2351638

**Published:** 2019-08-18

**Authors:** Pukovisa Prawiroharjo, Hainah Ellydar, Peter Pratama, Rizki Edmi Edison, Sitti Evangeline Imelda Suaidy, Nya' Zata Amani, Diavitri Carissima

**Affiliations:** ^1^Neurology Department, Faculty of Medicine Universitas Indonesia/Cipto Mangukusumo Hospital, Jakarta, Indonesia; ^2^Yayasan Kita Dan Buah Hati, Bekasi, Indonesia; ^3^Independent Scholar, Indonesia; ^4^Neuroscience Center-University of Muhammadiyah Prof. Dr. HAMKA, Jakarta, Indonesia

## Abstract

We aimed to find the differences in memory capabilities between pornography-addicted and nonaddicted juveniles. We enrolled 30 juveniles (12–16 y) consisting of 15 pornography addiction and 15 nonaddiction subjects. We used Rey Auditory Verbal Learning Test (RAVLT) to measure verbal memory, Rey–Osterrieth Complex Figure Test (ROCFT) for visual memory, along with Trail Making Test A and B (TMT-A and TMT-B) for attention. We found a significant reduction in the RAVLT A6 result of the addiction group (nonaddiction vs addiction: 13.47 ± 2.00 vs 11.67 ± 2.44, MD = −1.80, *p*=0.04), but not in ROCFT or attention tests. Analysis in sex subgroups yielded no sex-specific difference. We concluded that pornography addiction may be associated with impaired recent verbal memory in juveniles, regardless of sex and without association to attention.

## 1. Introduction

Substance addiction has since long been known to cause various cognitive and behavioral disorders, due to its direct effect on brain circuitry especially in the prefrontal cortex [1]. However, it has been proposed that behavioral addictions may also cause similar effects on the brain [2]. Among them, the Diagnostic and Statistical Manual of Mental Disorder Fifth Edition (DSM-5) by American Psychiatric Association in 2013 has recognized gambling disorder as official diagnosis and considered Internet gaming disorder for further study [2, 3]. However, pornography addiction was deemed as lacking research and remained unmentioned.

Trend in pornography becomes more prevalent among juveniles in this modern time as they are exposed to technology and Internet. Yayasan Kita Dan Buah Hati found that almost 97% of fourth to sixth-grade primary school students in Jakarta and its surrounding area have been exposed to pornographic contents from various forms of media [4]. This may significantly affect their social behavior, especially to sexual-related activity, potentially change the structure and activity of their brains, and may result in Internet pornography addiction. This, in turn, was associated with impaired cognitive functions, i.e., attention, working memory, and cognitive control [2], as were other behavioral addictions (e.g., pathological gambling [5, 6] and Internet addiction [7–10]), as was substance addiction itself [5, 11–15].

To the best of our knowledge, all previous studies regarding pornography addiction were performed on adult subjects. However, we believe it is also necessary to study the relationship between pornography addiction and cognitive function on those who are most vulnerable to it: juveniles, since it is the age group of brain maturation and is most vulnerable to addiction [16, 17]. This study aimed to appraise the differences in memory capabilities between pornography-addicted and nonaddicted juveniles.

## 2. Materials and Methods

### 2.1. Participants

A total of 30 juvenile subjects (aged 12–16 y) were screened using Pornography Addiction Test developed by Yayasan Kita Dan Buah Hati (explained below) to allocate them into pornography addiction group (*n*=15) and nonaddiction group (*n*=15). Pornography addiction is defined as test score equal or greater than 32. Enrollment was done during December 2017–February 2018, in various events held by YKBH in Bekasi, Indonesia. Exclusion criteria were left-handed, verbal or language disorder, history of brain-related disorder or disease, head trauma, trauma during pregnancy or birth, developmental, psychological, or neurological disorder, or mental illness.

### 2.2. Pornography Addiction Screening

To determine pornography addiction, we used a self-reported questionnaire developed by expert psychologists. Based on field studies and literature researches, we found several indicators commonly found in juveniles with high pornography consumption. The indicators can be grouped into three dimensions: (1) time spent to use pornography, defined as number of times, frequency, and duration spent to use pornography in the last six months; (2) motivation to use pornography, defined as factors encouraging access to pornography, such as sexual curiosity, emotional avoidance, sensation seeking, and sexual pleasure; and (3) problematic pornography use, defined as distress and functional problems, excessive use, control difficulties, and use of pornography to escape/avoid negative emotions. The questionnaire consisted of 92 items and has been tested on 740 students of grade six to ten in Indonesia, detailed in an unpublished report. To minimalize possibility of faking good, there were 3 additional questions; subjects who answered these according to social desire will be excluded. Psychometric analysis showed that all items are valid (CFA > 1.96) and reliable (Cronbach's alpha > 0.7). Pornography addiction was defined as weighted score of greater than or equal to 32.

The questionnaire was specially developed and adapted to juvenile population in the context of pornography; therefore, it was very suitable for this study. Additionally, it had a fail-safe mechanism from subjects who faked good, and most questions used forced choice technique which allows for less bias.

Limitation of this questionnaire included its number of questions, which may induce fatigue and boredom on the subjects. Additionally, its use in other context outside of juvenile pornography addiction may require wording adjustments, as knowledge of pornography-related vocabularies was crucial in understanding and responding to the questions.

### 2.3. Memory Assessments

To assess the participants' memory functions, we used the A6 and A7 scores of Ray Auditory Verbal Learning Test (RAVLT) for auditory-verbal memory, along with recall/delayed score of Ray–Osterrieth Complex Figure Test (ROCFT) for visual memory. Additionally, as attention has been widely recognized an important factor in working memory [18, 19], we also evaluated Trail Making Test (TMT) A and B. All tests were performed using standard procedures described in respective articles [20–23].

### 2.4. Ethical Approval

We did not expose our subjects to any form of pornography in all tests. The study was approved by Health Research Ethical Committee of Faculty of Medicine Universitas Indonesia (Clearance No. 1155/UN2.F1/ETIK/2017).

### 2.5. Statistical Analysis

The Mann–Whitney test was used for comparison between addiction and nonaddiction groups. We also compared memory assessment results between sex subgroups in each group. Statistical significance was assumed on *p* < 0.05. All statistical analyses were performed using SPSS® version 22 on Windows 7.

## 3. Results

### 3.1. Demographic Data

We enrolled 30 subjects (nonaddiction group vs addiction group: mean age = 13.27 ± 1.03 vs 13.80 ± 1.26 y) (Table 1). Both groups were age-matched (*p*=0.23).

### 3.2. Memory Assessment Results

**Figure 1:**
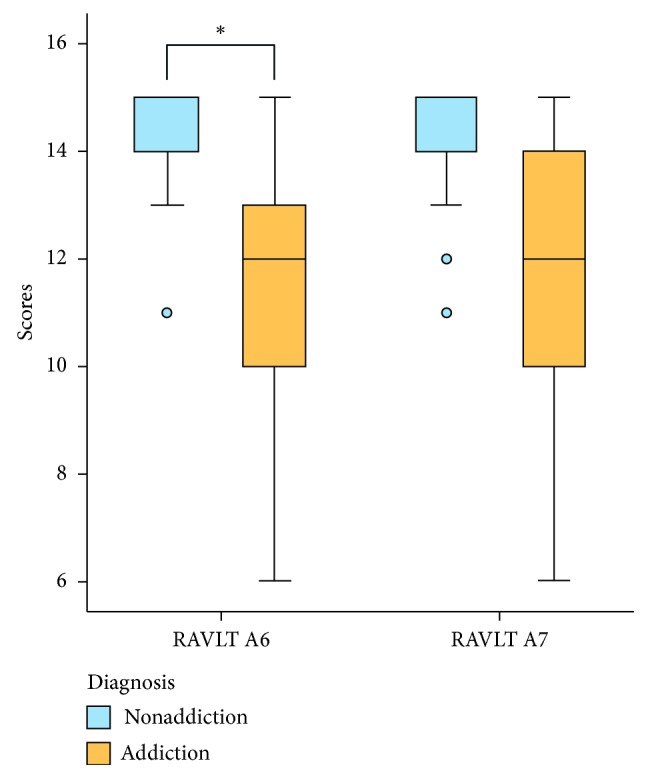
Box plot of RAVLT A6 and A7, compared between groups. ^*∗*^Statistically significant (*p* < 0.05).

There was significant difference between addiction and nonaddiction groups in RAVLT A6 (MD = −1.80, *p*=0.04), along with tendency, but not statistically significant, of difference in A7 (MD = −1.60, *p*=0.08) (Table 1, Figure 1). Further comparison in sex subgroups did not show sex-specific difference, apart from the tendency in RAVLT A7 on male subjects (MD = −2.30, *p*=0.07). There was no significant difference in ROCFT, TMT-A, and TMT-B test results.

## 4. Discussion

We found lower RAVLT A6 score in the pornography addiction group when compared to the nonaddiction group, by 1.80 point of mean difference (13.36% of nonaddiction score). As A6 signifies recent memory capability after disruption (in B1), our results showed diminishing memory capability on pornography addiction. Working memory is known to have an important role in maintaining goal-oriented behavior [24, 25]; therefore, our findings suggested that pornography-addicted juveniles may have problem to do so.

As this study was the first to specifically learn about memory function in pornography addiction, especially in juveniles, we were unable to directly compare with previous study. Therefore, we will attempt to discuss the results indirectly with other related studies, mainly Internet addiction, as both are behavioral-based addictions and the fact that many Internet addictions stem from using Internet to find pornographic materials [26].

An EEG study by Yu et al. on Internet addiction subjects found significantly decreased amplitude along with increased/delayed latency in P300 amplitudes when compared to nonaddiction subjects, suggesting reduced memory capability [9]. P300 is a positive peaking wave in EEG occurring at ±300 ms after a stimulus resolves a degree of uncertainty [27], which is proposed to be associated with memory and attention [28, 29]. Consistent with Yu et al.'s study, various other studies found similar results on substance addiction [28, 29], such as alcohol [30], cannabis [31], cocaine [32, 33], and opioid/heroin [33–35]. Additionally, P300 abnormality is also associated with antisocial personality disorder and impulsive behavior [30, 36].

Previous studies found lower working memory in substance addiction [5, 15, 37–39], but not pathological gambling [5, 15]. Nie et al. studied the performance of Internet addicts on verbal working memory when faced with related Internet materials; the study found that the subjects' memory function in 2-back task was slightly worse than normal control, but surprisingly, they performed better on internet-related material compared to internet-unrelated material [10]. Laier et al. specifically used pornographic contents and found significantly impaired visual working memory in pictorial 4-back task [40], although this study did not specifically evaluate addiction. Since RAVLT, which we used, measures verbal memory, similar to what was evaluated in Nie et al.'s study, our results were better compared to this study and similarly found reduction in memory capability.

Further analysis (based on sex subgroups) showed no sex-specific difference between female and male subgroups. Although it has been traditionally known that pornography affects males more than females [2, 41, 42], here we presented sex equality on association of pornography addiction with impaired memory capability. Therefore, problems with pornography addiction are not exclusive to males and that females should also be screened and treated for pornography addiction.

Despite attention being a confounding factor for memory performance [18, 19], we found that there was no significant difference in attention test results between both groups, suggesting that the impaired memory in pornography addiction was not related to attention problem. Further studies are warranted to understand the cause of this impairment.

Limitation of this study, which was also its strength, was our enrollment of juvenile subjects. Despite our aim to pioneer pornography addiction study in its earliest and most critical phase, juvenile brains are still growing and developing [43] and thus might compensate underlying brain impairment [44]. Furthermore, although it is a common approach to use related materials in order to gain better results, it was unfortunately unfavorable in our study as showing pornography to juveniles is considered unethical. Secondly, our study, being a cross-sectional design, was unable to find the cause-and-effect relationship between lower memory capability and pornography addiction. Another thing to consider is that we did not correct our results for multiple comparisons, as our study had only 3 actual variables to compare: auditory immediate memory (represented by RAVLT A6), auditory delayed memory (A7), and visual delayed memory (ROCFT delayed), which we considered as too few to cause a multiple-comparison false-discovery error. Other information in our results were all accompanying data displayed for completion purpose: RAVLT A1–5 were results of process toward A6 and A7, while TMT A and B were to rule out attention disorder.

Further neurocognitive studies regarding pornography effects on memory, attention, and other aspects of cognition, especially on longitudinal and functional imaging designs, are required to confirm the cause and extent of impairment.

## 5. Conclusions

Pornography addiction may be associated with impaired recent verbal memory in juveniles, regardless of sex and without association to attention.

## Figures and Tables

**Table 1:** Demographic and test score comparison.

	Nonaddiction (*n*=15)	Addiction (*n*=15)	MD	*p*
Sex (female : male)	7 : 8	5 : 10		
Age	13.27 ± 1.03	13.80 ± 1.26	0.53	0.23
RAVLT A1	6.87 ± 2.77	6.87 ± 2.20	0.00	0.82
RAVLT A2	9.33 ± 2.53	9.13 ± 2.77	−0.20	0.93
RAVLT A3	11.47 ± 3.40	12.00 ± 2.65	0.53	0.83
RAVLT A4	13.07 ± 1.94	13.20 ± 2.24	0.13	0.70
RAVLT A5	13.27 ± 2.25	14.27 ± 1.16	1.00	0.26
RAVLT B1	7.73 ± 2.91	7.47 ± 2.53	−0.26	0.95
RAVLT A6	13.47 ± 2.00	11.67 ± 2.44	−1.80	0.04^*∗*^
RAVLT A7	13.67 ± 1.76	12.07 ± 2.63	−1.60	0.08
ROCFT recall	24.10 ± 4.79	23.23 ± 6.57	−0.87	0.88
TMT-A (second)	40.20 ± 13.13	44.67 ± 14.49	4.47	0.40
TMT-B (second)	93.27 ± 30.89	91.60 ± 53.48	−1.67	0.58

^*∗*^Statistically significant (*p* < 0.05). All values are in mean ± SD, except stated. MD = mean difference (addiction − nonaddiction); RAVLT = Rey Auditory Verbal Learning Test; ROCFT = Rey–Osterrieth Complex Figure Test.

## Data Availability

The memory performance measurement score data used to support the findings of this study are included within the article.

## Supplementary Materials

Supplementary Materials Comparison of memory and attention test scores between nonaddiction and addiction groups, subgrouped by sex.
