# Supplementary material for: Impaired Recent Verbal Memory in Pornography-Addicted Juvenile Subjects
Source: Neurol Res Int. 2019 Aug 18;2019:2351638. doi: 10.1155/2019/2351638 (PMC6721264; doi:10.1155/2019/2351638)
Supplement: Supplementary Materials — Comparison of memory and attention test scores between nonaddiction and addiction groups, subgrouped by sex. [file 2351638.f1.pdf]

Supplementary Table 1. Comparison of memory and attention test scores between non-addiction and addiction group, subgrouped by sex

		Non-Addiction (n = 15)	Addiction (n = 15)	p
**Age**	Female	13.00 ± 1.00	13.83 ± 1.33	0.25
	Male	13.50 ± 1.07	13.78 ± 1.30	0.62
**RAVLT A1**	Female	5.71 ± 2.69	6.17 ± 1.72	0.43
	Male	7.88 ± 2.59	7.33 ± 2.45	0.66
**RAVLT A2**	Female	7.71 ± 0.95	6.83 ± 2.14	0.41
	Male	10.75 ± 2.66	10.67 ± 2.00	0.73
**RAVLT A3**	Female	11.14 ± 3.67	10.50 ± 2.88	0.61
	Male	11.75 ± 3.37	13.00 ± 2.06	0.46
**RAVLT A4**	Female	12.86 ± 1.77	12.00 ± 2.97	0.61
	Male	13.25 ± 2.19	14.00 ± 1.22	0.61
**RAVLT A5**	Female	12.71 ± 2.36	13.67 ± 1.51	0.45
	Male	13.75 ± 2.19	14.67 ± 0.71	0.40
**RAVLT B1**	Female	8.43 ± 2.57	7.17 ± 3.06	0.52
	Male	7.13 ± 3.23	7.67 ± 2.29	0.56
**RAVLT A6**	Female	13.57 ± 1.81	12.33 ± 1.86	0.27
	Male	13.38 ± 2.26	11.22 ± 2.77	0.12
**RAVLT A7**	Female	13.71 ± 1.38	13.17 ± 2.14	0.82
	Male	13.63 ± 2.13	11.33 ± 2.78	0.07
**ROCFT**	Female	24.50 ± 3.71	24.00 ± 3.74	0.89
	Male	23.75 ± 5.81	22.72 ± 8.12	1.00
**TMT A**	Female	41.14 ± 12.51	50.00 ± 17.55	0.20
	Male	39.38 ± 14.45	41.11 ± 11.82	0.85
**TMT B**	Female	108.00 ± 29.98	102.33 ± 84.84	0.20
	Male	80.38 ± 27.05	84.44 ± 19.06	0.19

* statistically significant ($p < 0.05$)
All values are in mean ± SD, except stated.
MD = Mean Difference (addiction - non-addiction); RAVLT = Rey Auditory Verbal Learning Test; ROCFT = Rey-Osterrieth Complex Figure test
